# Continued breathing followed by gasping or apnea in a swine model of ventricular fibrillation cardiac arrest

**DOI:** 10.1186/1471-2261-10-36

**Published:** 2010-08-09

**Authors:** Mathias Zuercher, Gordon A Ewy, Ronald W Hilwig, Arthur B Sanders, Charles W Otto, Robert A Berg, Karl B Kern

**Affiliations:** 1 University of Arizona Sarver Heart Center, Tucson, AZ, USA; 2 Department of Anesthesia and Intensive Care Medicine, University Hospital Basel, Basel, Switzerland; 3 Department of Medicine, University of Arizona College of Medicine, Tucson, AZ, USA; 4 Department of Emergency Medicine, University of Arizona College of Medicine, Tucson, AZ, USA; 5 Department of Anesthesiology, University of Arizona College of Medicine, Tucson, AZ, USA; 6 Department of Anesthesia and Critical Care, The Children's Hospital of Philadelphia, Philadelphia, PA, USA

## Abstract

**Background:**

Continued breathing following ventricular fibrillation has here-to-fore not been described.

**Methods:**

We analyzed the spontaneous ventilatory activity during the first several minutes of ventricular fibrillation (VF) in our isoflurane anesthesized swine model of out-of-hospital cardiac arrest. The frequency and type of ventilatory activity was monitored by pneumotachometer and main stream infrared capnometer and analyzed in 61 swine during the first 3 to 6 minutes of untreated VF.

**Results:**

During the first minute of VF, the air flow pattern in all 61 swine was similar to those recorded during regular spontaneous breathing during anesthesia and was clearly different from the patterns of gasping. The average rate of continued breathing during the first minute of untreated VF was 10 breaths per minute. During the second minute of untreated VF, spontaneous breathing activity either stopped or became typical of gasping. During minutes 2 to 5 of untreated VF, most animals exhibited very slow spontaneous ventilatory activity with a pattern typical of gasping; and the pattern of gasping was crescendo-decrescendo, as has been previously reported. In the absence of therapy, all ventilatory activity stopped 6 minutes after VF cardiac arrest.

**Conclusion:**

In our swine model of VF cardiac arrest, we documented that normal breathing continued for the first minute following cardiac arrest.

## Background

Gasping following cardiac arrest is a well-investigated phenomenon [1-10]. Gasping probably occurs as a response to decreased brain perfusion and/or to hypoxia [11]. It has been observed in all mammals investigated, may be present at birth and at death, and is considered an "auto-resuscitative" phenomenon [12-14]

Gasping is an important phenomenon in humans suffering from cardiac arrest, with a reported occurrence of 55% in patients with witnessed out-of-hospital cardiac arrest [1]. Survival has been reported in 39% of humans with witnessed arrest who gasped while receiving bystander resuscitation efforts but in only 9% of individuals who did not gasp while receiving bystander resuscitation efforts [2].

Spontaneous ventilatory activity consistent with continued breathing during the first minutes of VF cardiac arrest has not been previously described. We observed this phenomenon in swine and performed ventilation recordings to characterize the ventilatory patterns.

## Methods

This study was conducted with the approval of the University of Arizona Institutional Animal Care and Use Committee in accordance with the guidelines set forth in the "Position of the American Heart Association on Research Animal Use". This report is an analysis of the respiratory patterns following VF cardiac arrest of a previously reported study comparing two basic resuscitation protocols with regard to 24-hr neurological outcome [15]. Sixty-four swine (28 ± 4 kg) were anesthetized by inhalation of 5% isoflurane in oxygen. An endotracheal tube was placed, and anesthesia was maintained using 1.5 - 3% isoflurane in ambient air. Ventilation was provided by a rate- and volume-regulated ventilator (Narkomed 2A, North American Drager, Telford, PA, USA) and was adjusted to maintain an end-tidal CO_2 _pressure (EtCO_2_) of 40 ± 3 mmHg. Vascular introducer sheaths (7F, Cordis Corp., Miami, FL, USA) were placed by sterile cutdown procedures, and solid state pressure transducers (MPC-500, Millar Instruments, Houston, TX, USA) were positioned into the descending aorta and the right atrium. Electrocardiographic leads were placed on the limbs. An infrared capnometer (47210A, Hewlett Packard Co., Palo Alto, CA, USA) and a pneumotachometer (MP45-871, Validyne Engineering Corp., Northridge, CA, USA) were used to measure EtCO_2 _and air flow, respectively. Defibrillator pads (Quik-Combo, Medtronic, PhysioControl, Redmond, WA, USA) were adhered to the chest. All mentioned parameters were continuously displayed on a recording system (Gould Ponemah Physiology Platform, Model P3 Plus, LDS Life Science, Valley View, OH, USA) and stored on a laptop computer for analysis. After collection of baseline data, VF was induced by alternate current via a pacing electrode temporarily placed in the right ventricle. VF was confirmed by the fall in the arterial blood pressure and the electrocardiographic pattern. Assisted ventilation was discontinued immediately after induction of VF. Two additional control swine were similarly investigated, but rather than assisted ventilation, a period of spontaneous breathing during isoflurane anesthesia was allowed prior to the induction of VF to determine the normal breathing pattern.

Breathing was defined as a ventilatory pattern similar to that recorded during spontaneous ventilation under isoflurane anesthesia. Gasping has been previously defined as an abrupt transient inspiratory effort [16]. To avoid over-interpretation of irrelevant signals or artifacts, due to the high sensitivity of the capnometer, we defined any short inspiratory air flow followed by a rapid decrease of at least 25% of the EtCO_2 _as a gasp. Data on gasping was available in 61 of the 64 swine. Of those with no data, the capnograph malfunctioned in one animal, and in two others the data files were missing.

### Statistical analysis

Data were entered into Microsoft Excel for Windows 2003 (Microsoft Corp, Redmond, WA, USA) and were analyzed using SPSS 16.0 for Windows (SPSS, Inc, Chicago, IL, USA). Continuous variables were presented as mean ± SD and median.

## Results

**Figure 1:**
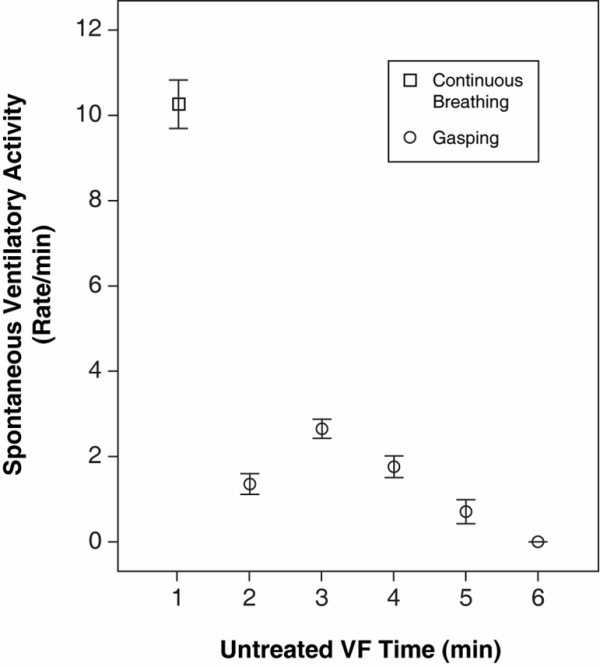
**Spontaneous ventilatory activity during the first 6 minutes of untreated ventricular fibrillation (VF) plotted as mean ± standard error of the mean (SEM) for each minute after induction of VF**.

The baseline data are presented in Table 1. All 61 animals exhibited continuous spontaneous ventilatory activity during the first minute following the induction of VF (Figure 1) at an average rate of 10 breaths per minute (Figure 1, Table 2). During minutes 2 to 5, the occurrence of gasping varied between 23% and 82%, and the frequency was less than 3 gasps per minute (Table 2).

**Table 1:** Baseline data

Gender, female/male	26/35
Weights, kg	28 ± 4
Heart rate, bpm	105 ± 16
Mean systolic BP, mmHg	81 ± 15
Mean diastolic BP, mmHg	55 ± 11
Cardiac output, L/min	2.5 ± 0.5
SaO_2, _%	95 ± 3
PaO_2_, mmHg	77 ± 18
PaCO_2_, mmHg	42 ± 3

**Table 2:** Occurrence and frequency of spontaneous ventilatory activity during untreated VF

VF time	Occurrence		Frequency	
(min)	*n/N*	%	Mean (SD)	Median
1	61/61	100%	10.3 (4.4)	10
2	33/61	54%	1.4 (1.8)	1
3	50/61	82%	2.6 (1.8)	3
4	30/46	65%	1.8 (1.7)	2
5	7/31	23%	0.7 (1.6)	0
6	0/16	0%	0 (0)	0

*n *= number of animals experiencing gasping. *N *= total number of animals in the respective VF time group. Frequency = number of spontaneous ventilations or gasps per minute.

**Figure 2:**
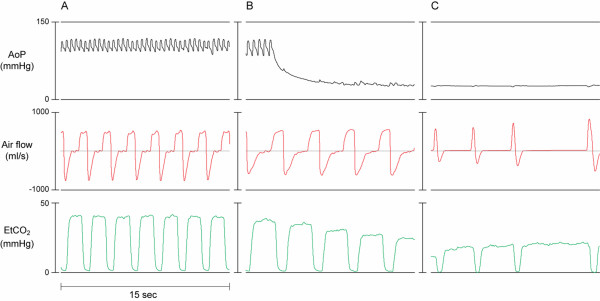
**Graphic recording of aortic pressure (AoP), air flow signal, and end-tidal CO_2 _(EtCO_2_) curves in anesthetized swine during**: A) Spontaneous breathing during normal sinus rhythm, B) Spontaneous breathing following the induction of ventricular fibrillation (VF), and C) Gasping during untreated VF. Note the relative plateau in the inspiratory air flow of the spontaneous breaths compared with the sharp "peak" in the air flow of the gasps.

**Figure 3:**
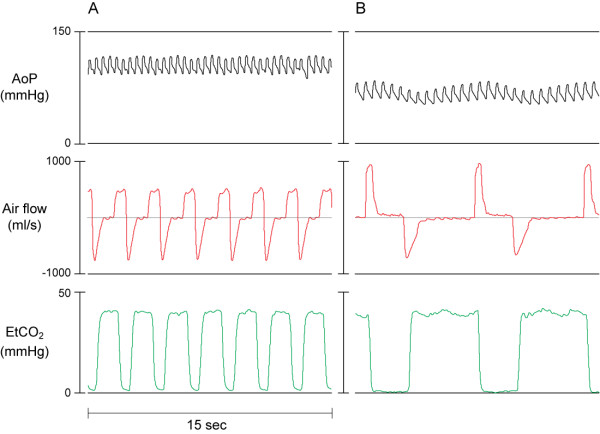
**Graphic recording showing the aortic pressure (AoP), air flow signal and end-tidal CO_2 _(EtCO_2_) curves in anesthetized swine during**: A) Spontaneous breathing during normal sinus rhythm. B) Mechanical ventilation.

**Figure 4:**
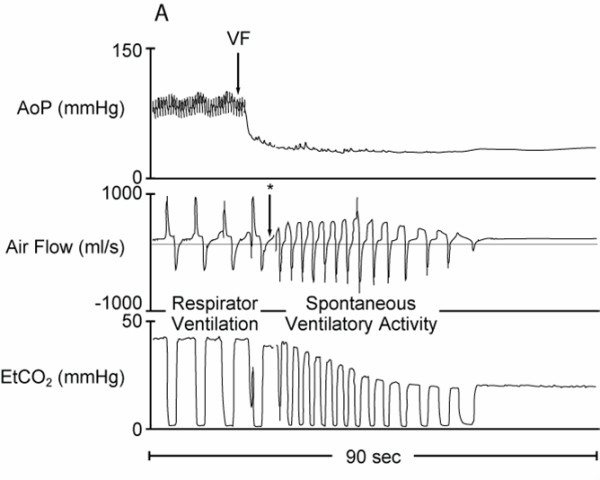
**Graphic recording showing the aortic pressure (AoP), air flow signal and end-tidal CO_2 _(EtCO_2_) curves in anesthetized swine during the first 90 seconds of VF cardiac arrest**. The asterisk (*) indicates the time point when the endotracheal tube was disconnected from the mechanical ventilator.

The air flow pattern during the initial minute after induction of VF appeared to be very similar to the flow pattern of normal spontaneous ventilation during anesthesia and was distinctly different from gasping (Figure 2). This spontaneous ventilation pattern was characterized by an initial increase in inspiratory flow reaching a plateau followed by high flow expiration (Figure 2A, B). This pattern is different from that observed during gasping (Figure 2C). The air flow pattern of typical gasps consists of a rapid increase in inspiratory flow, which immediately changes after reaching peak values, to a peak expiratory flow and a subsequent pause (Figure 3). The airflow pattern of breathing was also different from that of mechanical assisted ventilation (Figure 3 and 4). In contrast, the air flow pattern of typical gasps consists of a rapid increase in inspiratory flow, which immediately changes to a peak expiratory flow and a subsequent pause after reaching peak values (Figure 2C).

## Discussion

In this swine model of out-of-hospital cardiac arrest, our group observed that following the induction of VF, all animals continued breathing for about one minute at a mean frequency of 10 breaths per minute (Figure 1). The continued breathing was then followed by the crescendo-decrescendo pattern of gasping that has previously been described [8] (Figure 1). The recorded air flow pattern during the first minutes following the onset of ventricular fibrillation was similar to that of regular spontaneous breathing of an anesthetized animal and was different from the air flow pattern typical for gasping or agonal ventilation that occurred during minutes 2 to 5 (Figure 2). This continued breathing pattern was also clearly different from the pattern of assisted mechanical ventilation (Figure 3). The finding of continued breathing for the first minute following VF cardiac arrest, if confirmed in humans, is of clinical relevance. The failure to recognize spontaneous breathing and gasping following cardiac arrest potentially delays the recognition of the arrest and, thus, the early initiation of resuscitation efforts.

The explanation for the observation of continued breathing during the first minute following VF cardiac arrest might well be related to carotid artery and cerebral blood flow patterns immediately following untreated VF cardiac arrest. Frenneaux and Steen [17,18] reported that carotid artery blood flow was relatively well maintained for the first minute following untreated VF cardiac arrest, before rapidly and markedly decreasing during the second minute and remaining marginal but still present for up to four minutes. Ristagno and colleagues [8] also found that there was substantial cerebral cortical microvascular flow after the onset of VF that rapidly decreased during the first three minutes of untreated VF cardiac arrest in swine. Cerebral blood flow differs from coronary artery blood flow, which ceases almost immediately after VF cardiac arrest [18].

Our observation of normal breathing during the first minute of VF was followed by a slower rate of spontaneous ventilation during minutes 2 to 5, which was typical of gasping or agonal respiration. As shown in Figure 2, gasping has a ventilatory flow pattern distinctly different from normal breathing or mechanical ventilation and typically exhibits a crescendo-decrescendo frequency pattern. After the fifth minute of untreated VF, gasping was not observed.

Gasping following cardiac arrest has been previously reported in human [1-3] as well as in animal models. In 1988 study in rats, von Planta and colleagues [19] reported that "gasping typically began 1 min after the induction of VF". In a swine model of VF cardiac arrest, Srinivasan [9] found that the time to the first gasp was 1.5 ± 0.5 min and that gasping ended on average 4.7 min after the onset of VF cardiac arrest. Ristagno and colleagues [8] also reported on the frequency of gasping during minutes 2 to 5 after onset of VF cardiac arrest. In their swine model, Frenneaux and Steen [18] found that gasping typically began weakly, increased to a maximum and generally ceased entirely by about 5 minutes post VF induction. These reports do not comment on spontaneous ventilatory activity during the first minute of VF cardiac arrest.

Menegazzi et al. [20] reported in a study of 12 swine that were sedated with ketamine/xylazine, and anesthetized with alpha-chloralose that all had agonal respirations through the first 2 minutes of arrest. The number of swine presenting spontaneous respiratory activity decreased to 11 (92%) at minute 3, five (42%) at minute 4, and two (17%) at minute 7. Mean respiratory rates ranged from 6 to 11 breaths/min. This report did not analyze the air the air flow pattern or the wave form of the end-tidal CO_2 _curves and invariably refers to post arrest ventilations as agonal respirations, whereas our findings suggest two different types of agonal respirations; initially continued breathing followed by typical agonal or gasping ventilations.

We believe that this is the first published report showing two different spontaneous air flow patterns during the early phase following VF cardiac arrest. However, based upon the references cited we wonder whether this has been a present but unrecognized phenomenon.

The pattern of continued spontaneous breathing for the first minute of VF cardiac arrest may be explained by continued perfusion through the carotid artery (and presumably continued cerebral blood flow), as reported by Frenneaux and Steen [18] following the induction of VF. Breathing no doubt stops in response to inadequate cerebral blood flow. Gasping, produced by a more primitive ventilatory center located in the brain stem is then activated [11-14]. Gasping is initiated during the second minute of VF cardiac arrest and increases during the third minute, but as the gasping center in the brain stem becomes more ischemic, the frequency of gasping decreases and eventually stops. This is a plausible explanation for the classically described crescendo-decrescendo pattern of gasping following untreated cardiac arrest [18].

There are limitations to this study that must be considered. This study is observational only and was not designed to determine the mechanisms of spontaneous breathing and gasping. Gasping is markedly more pronounced in immature animals [11-13]. Although the young swine used in this study were not immature, they might have exhibited a higher relative frequency of gasping compared with aged animals. The phenomenon of gasping is also related to the type and duration of anesthesia the animals are subjected to before the induction VF and the presence or absence of paralysis. The swine in this study were not paralyzed and were maintained on a relatively low concentration of isoflurane, which is less likely to suppress gasping relative to some other commonly used anesthetics, but might reduce the respiratory stimulation due to increasing pCO_2 _[21]. In our experience, high doses of isoflurane will also suppress gasping.

## Conclusions

In this swine model of VF cardiac arrest, spontaneous ventilatory activity was observed in all 61 animals during the first minute after cardiac arrest. The airflow pattern of ventilations during the first minute was the same as that exhibited during spontaneous breathing in anesthetized swine. Thereafter, the airflow pattern changed to that typical of gasping. This pattern change is suggestive of a shift in the "respiratory center" from the breathing center located in the medullo-pontine area caudally to a gasping center located in the medulla oblongata. Gasping was not present after 5 minutes of untreated VF.

Spontaneous ventilatory activity following VF cardiac arrest is a potentially important phenomenon. The observation of continued breathing following VF cardiac arrest is reported to encourage others to determine whether this phenomenon also occurs in man. If so, these phenomena may well have important clinical implications for the early recognition of cardiac arrest.

## Key messages

• In swine undergoing VF cardiac arrest, normal spontaneous breathing occurred during the first minute.

• Gasping was observed in swine between minutes 2 and 5, but never after minute 5, of untreated VF cardiac arrest.

• If found to occur in man, spontaneous ventilatory activity following VF cardiac arrest may have important implications for the early recognition of cardiac arrest.

## Abbreviations

AOP: aortic pressure; ETCO_2_: end-tidal CO_2_; SEM: standard error of the mean; VF: ventricular fibrillation;

## Competing interests

The authors declare that they have no competing interests.

## Authors' contributions

This study was conceived and designed by MZ, GAE, RWH, RAB and KBK. MZ, GAE, RWH, ABS, CWO and KBK analyzed and interpreted the data. MZ conducted the statistical analysis, and MZ and GAE drafted the original version of the manuscript. MZ, GAE, RWH, ABS, CWO and KBK revised the manuscript critically for important intellectual content and share responsibility for the final approval of the manuscript. All authors have read and approved the final manuscript.

## Pre-publication history

The pre-publication history for this paper can be accessed here:

http://www.biomedcentral.com/1471-2261/10/36/prepub
